# Valgus Osteotomy With Dynamic Hip Screw (DHS) Fixation for Nonunion and Malunion of Trochanteric Fractures: A Single-Center Experience

**DOI:** 10.7759/cureus.84277

**Published:** 2025-05-17

**Authors:** Farman Ul Haq, Kamran Sabir, Hazrat Akbar, Ziarmal Khan, Abid Ullah, Rehmat Khan

**Affiliations:** 1 Department of Orthopaedics, Ghurki Trust Teaching Hospital, Lahore, PAK; 2 Department of Orthopaedics, Hayatabad Medical Complex Peshawar, Peshawar, PAK

**Keywords:** dynamic hip screw fixation, harris hip score, intertrochanteric fracture, nonunion, valgus corrective osteotomy, varus malunion

## Abstract

Objective

The objective of this study was to determine the functional of valgus osteotomy with dynamic hip screw (DHS) fixation in managing patients diagnosed with varus malunion and non-union trochanteric fractures.

Methodology

This descriptive case series was conducted at the Department of Orthopaedics and Spine Centre, Ghurki Trust Teaching Hospital, Lahore, Pakistan, from June 7, 2023, to December 7, 2023. A total of 80 patients aged above 40 years with malunited or non-united trochanteric fractures were included using non-probability consecutive sampling. Patients with active hip infections or lost to follow-up were excluded. Ethical approval was obtained, and informed consent was taken. All patients underwent valgus osteotomy under spinal anesthesia, followed by fixation with a DHS. Postoperative follow-ups were conducted at two and three months, with functional outcomes assessed using the Harris Hip Score (HHS) at three months. Data were analyzed using IBM SPSS Statistics for Windows, Version 23 (Released 2015; IBM Corp., Armonk, New York, United States), with p ≤ 0.05 considered statistically significant.

Results

The study included 80 patients with a mean age of 54.83 ± 10.41 years, predominantly male (85.0%). Falls were the most common mode of injury (n=48, 60.0%). The mean HHS at three months postoperatively was 84.74 ± 8.06, with 37.5% (n=30) of patients achieving excellent outcomes, 40.0% (n=32) good, 17.5% (n=14) fair, and 5% (n=4) poor. Stratification by gender, age, and mode of injury showed no statistically significant differences in outcomes (p > 0.05).

Conclusion

Valgus osteotomy with DHS fixation demonstrated good outcomes in the majority of participants, followed by excellent outcomes. While female and younger patients showed slightly better results, these differences were not statistically significant. Falls were the most common cause of injury, and the procedure achieved high success rates regardless of the mode of injury. These findings support valgus osteotomy with DHS fixation as a reliable treatment for nonunion and malunion trochanteric fractures.

## Introduction

Trochanteric fractures account for approximately 45-50% of all hip fractures in older adults, with 50-60% being unstable [1,2]. These fractures are common in the elderly due to poor bone stock, while in younger individuals, they often result from high-velocity trauma such as falls from a height or road traffic accidents [1,2]. Patients with these fractures experience significant functional limitations, including difficulty squatting and sitting cross-legged, which are essential for daily activities like defecation and eating. This disability often leads patients to seek treatment at a later stage, further complicating management [3].

The ideal treatment for failed and malunited intertrochanteric fractures has traditionally been total hip arthroplasty [3]. However, this procedure is expensive and often unsuitable for patients from rural populations who cannot afford it or require the ability to squat and sit cross-legged [3]. A review of the literature reveals a lack of alternative procedures for managing these malunited fractures. There is a need for a cost-effective method that restores limb length, corrects the neck-shaft angle, improves hip range of motion, and enables squatting and cross-legged sitting while preserving the abductor mechanism [2,3].

Valgus osteotomy with dynamic hip screw (DHS) fixation is a promising solution. Although traditionally used for femoral neck fractures in younger populations to salvage the femoral head, it has not been widely described for managing malunited intertrochanteric fractures [4]. This procedure addresses limb length discrepancy, restores the neck-shaft angle, improves hip mobility, and preserves the abductor mechanism, offering good functional outcomes [5,6].

Varghese et al. conducted a study on valgus osteotomy with DHS fixation for neglected femoral neck fractures, reporting excellent outcomes in 21.43% of cases, good in 32.14%, fair in 32.14%, and poor in 14.29% based on the Harris Hip Score (HHS) [6]. Similarly, other studies have shown good to excellent outcomes in patients with varus malunion and non-union trochanteric fractures [5,7].

In Pakistan, there is a lack of local studies on this topic. This study aims to provide evidence-based insights into the management of intertrochanteric fractures using valgus osteotomy with DHS fixation, with the goal of improving union rates and reducing complications. The objective of this study was to evaluate the functional outcome of valgus osteotomy with DHS in managing patients diagnosed with varus malunion and nonunion trochanteric fractures.

## Materials and methods

A descriptive case series was conducted at the Department of Orthopaedics & Spine Centre, Ghurki Trust Teaching Hospital, Lahore, Pakistan, over a six-month period from June 7, 2023, to December 7, 2023. The study was approved by the Ethical Review Committee, Ghurki Trust and Teaching Hospital (registration number: 2023/01/R-15), and informed consent was taken from all participants. 

Participants

A total of 80 patients were included in the study, with the sample size calculated using a 0.05 level of significance, a 9% margin of error, and an expected proportion of excellent outcomes of 21.43% [6]. Patients were selected through non-probability consecutive sampling based on predefined inclusion and exclusion criteria.

Inclusion Criteria

Patients aged above 40 years with malunion or nonunion hip trochanteric fractures were included.

Exclusion Criteria

Those with active hip infections, as determined through clinical assessment and laboratory investigations, and those lost to follow-up were excluded.

Procedure

**Figure 1:**
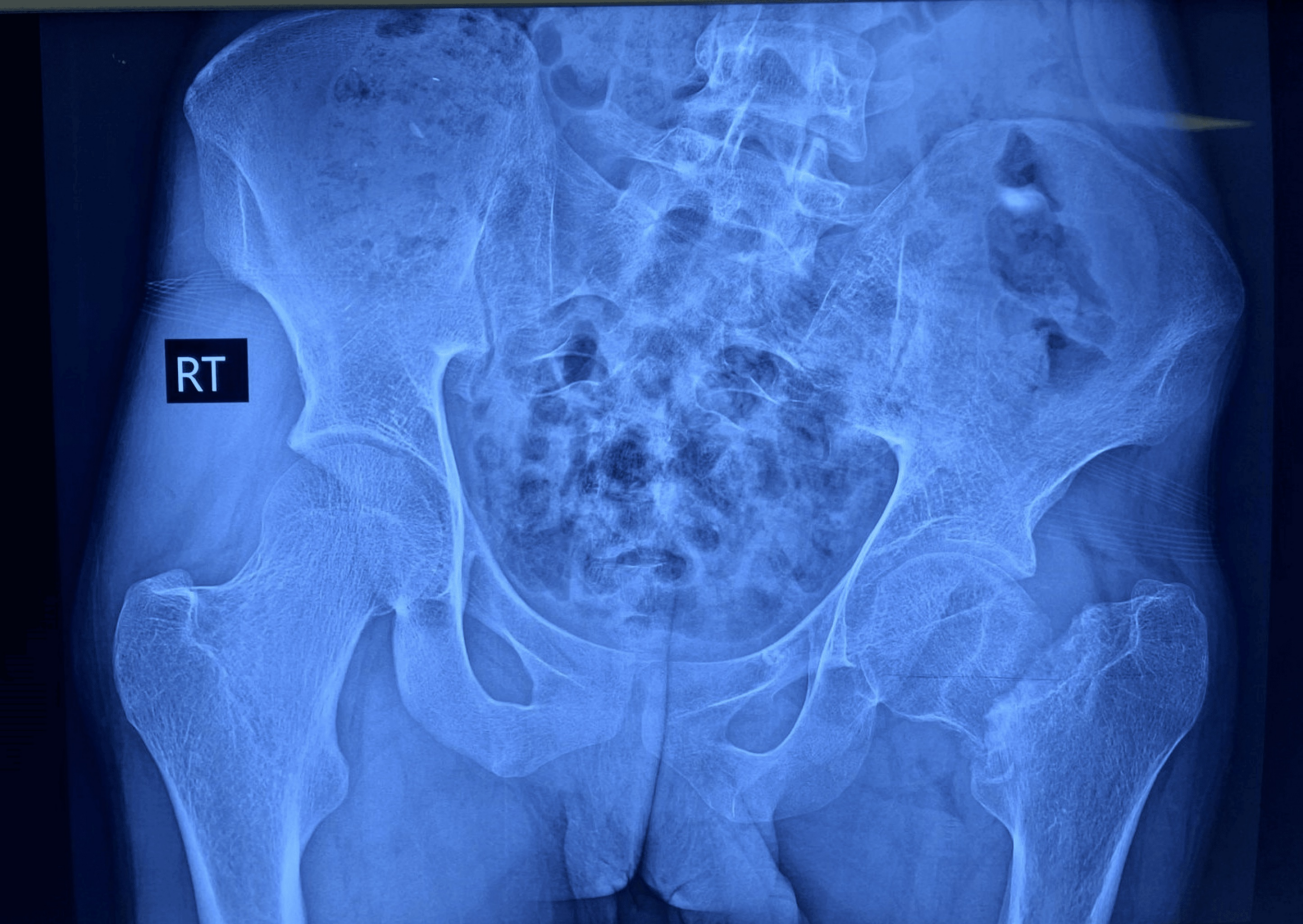
Preoperative X-ray image of a patient

**Figure 2:**
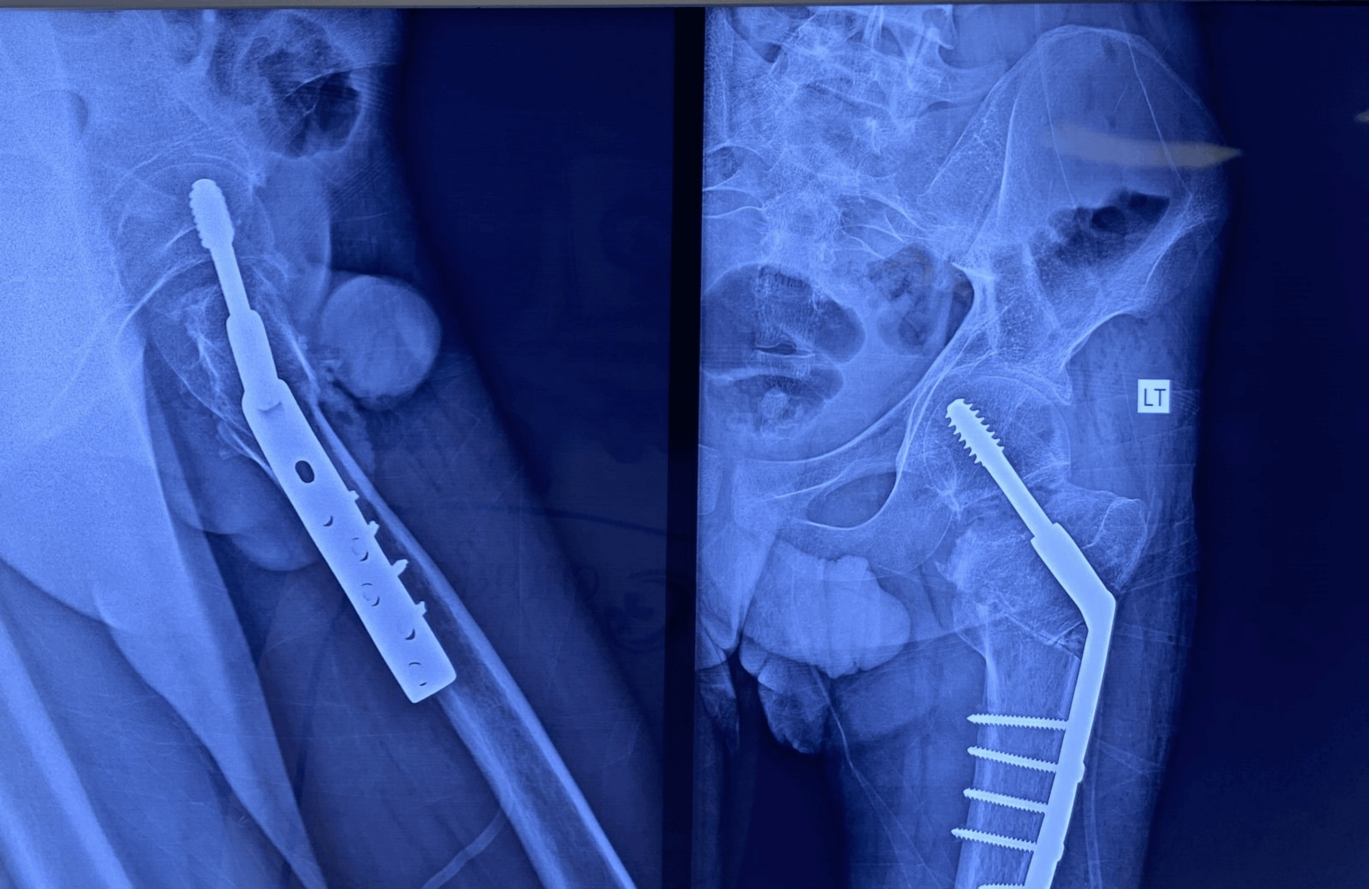
Postoperative X-ray image of a patient

All included patients underwent valgus osteotomy under spinal anesthesia using a standard lateral approach to expose the proximal femur. A 3.5 mm drill bit was used to create an entry point, and a guide wire was passed into the center of the femoral neck, with its position confirmed on anteroposterior and lateral views. An oblique osteotomy was performed distal to the lesser trochanter using an oscillating saw, followed by deformity correction and fixation with a DHS plate. The limb was returned to a neutral position to ensure proper restoration of limb length and neck-shaft angle. Hemostasis was achieved, a drain was placed, and the wound was closed in layers with a sterile dressing. Preoperative and postoperative images of a patient are shown in Figures 1, 2.

Postoperatively, patients underwent a standardized rehabilitation protocol, including early mobilization with weight-bearing as tolerated, and were followed up during routine outpatient visits at two and three months, with functional assessment at the three-month follow-up using the HHS. 

The HHS is a standardized tool for evaluating hip function, with higher scores indicating better outcomes. The maximum possible score was 100, categorized as poor (<70), fair (70-79), good (80-89), and excellent (90-100). Data were recorded using a predesigned questionnaire, including demographic details such as name, age, gender, address, hospital/ward number, and contact number, along with clinical outcomes.

Statistical analysis

Data analysis was performed using IBM SPSS Statistics for Windows, Version 23 (Released 2015; IBM Corp., Armonk, New York, United States). Continuous variables, such as age and HHS, were expressed as means and standard deviations, while categorical variables, including gender, mechanism of injury, and outcome classification, were reported as frequencies and percentages. Data were stratified for age, gender, and mechanism of injury to assess effect modification, and a post-stratification chi-square test was applied. A p-value ≤ 0.05 was considered statistically significant.

## Results

A total of 80 patients with nonunion and malunion of trochanteric fractures who underwent valgus osteotomy with DHS were included in the study. The majority of the patients were male (n=68, 85.0%), while females constituted 15.0% (n=12) of the study population. The mean age of the patients was 54.83 ± 10.41 years, ranging from 41 to 80 years. Regarding the mode of injury, 48 (60.0%) cases were due to falls, followed by 23 (28.7%) resulting from road traffic accidents (RTA) and nine (11.3%) from other causes. The mean HHS at three months postoperatively was 84.74 ± 8.06, with a minimum score of 67 and a maximum of 95. In terms of functional outcomes, 30 (37.5%) patients achieved an excellent result, 32 (40.0%) had a good outcome, 14 (17.5%) showed a fair outcome, and four (5.0%) had a poor outcome (Table 1).

**Table 1:** Demographic and clinical characteristics of study participants (N=80) Data has been presented as frequency (percentage) except for age and Harris Hip Score, which have been presented as mean±SD (range)

Variable	Category	Values
Gender, n (%)	Male	68 (85%)
	Female	12 (15%)
Age (years), mean±SD (range)	-	54.83±10.41 (41-80)
Mode of Injury, n (%)	Road Traffic Accident	23 (28.70%)
	Fall	48 (60%)
	Others	9 (11.30%)
Harris Hip Score, mean±SD (range)	Three months postoperative	84.74±8.06 (67-95)
Functional Outcome, n (%)	Excellent	30 (37.50%)
	Good	32 (40%)
	Fair	14 (17.50%)
	Poor	4 (5%)

When stratified by gender, six (50.0%) female patients had excellent outcomes compared to 24 (35.3%) of male patients. However, the chi-square test did not show a statistically significant difference (p = 0.576). Regarding age, patients aged 41-60 years had a higher proportion of excellent (n=21, 38.9%) and good (n=22, 40.7%) outcomes than those aged 61-80 years, where nine (30.0%) had excellent and 10 (33.3%) had good outcomes; however, the difference was not statistically significant (p = 0.239). Stratification by mode of injury revealed that patients with RTA had a higher percentage of good outcomes (n=13, 56.5%), whereas those with fall-related fractures had the highest proportion of excellent outcomes (n=20, 41.7%). Among patients with injuries due to other causes, four (44.4%) had excellent outcomes, while three (33.3%) had good outcomes. The chi-square test did not indicate a statistically significant association (p = 0.474) (Table 2).

**Table 2:** Association of gender, age, and mode of injury with functional outcomes RTA: road traffic accident

Variable	Category	Excellent	Good	Fair	Poor	Chi-Square value (P-value)
Gender	Male	24 (35.3%)	27 (39.7%)	13 (19.1%)	4 (5.9%)	1.928 (0.576)
	Female	6 (50.0%)	5 (41.7%)	1 (8.3%)	0 (0.0%)	
Age (years)	41-60	21 (38.9%)	22 (40.7%)	11 (20.4%)	1 (1.9%)	4.214 (0.239)
	61-80	9 (30.0%)	10 (33.3%)	3 (10.0%)	3 (10.0%)	
Mode of Injury	RTA	6 (26.1%)	13 (56.5%)	4 (17.4%)	0 (0.0%)	5.565 (0.474)
	Fall	20 (41.7%)	16 (33.3%)	9 (18.8%)	3 (6.3%)	
	Others	4 (44.4%)	3 (33.3%)	1 (11.1%)	1 (11.1%)	
Total		30 (37.5%)	32 (40.0%)	14 (17.5%)	4 (5.0%)	

## Discussion

Fresh intertrochanteric fractures are relatively straightforward to treat, with various implants and treatment options available. However, when these fractures are not managed properly, patients often present with malunion, coxa vara, and associated complications such as limb shortening, weakened abductor mechanisms, and restricted hip mobility. THA remains the gold standard for treating such cases, but it is often unsuitable for patients who require the ability to squat or sit cross-legged, particularly in rural populations [8,9]. Valgus osteotomy with DHS fixation offers a viable alternative, addressing limb length discrepancy, restoring the neck-shaft angle, and improving hip function without the need for extensive dissection or prolonged surgical time [10].

In the current study, 80 patients with malunion or nonunion trochanteric fractures were analyzed, with a mean age of 54.83 ± 10.41 years. The mean HHS was 84.74 at a follow-up visit. A study by Subash Y in 2020 also reported improvement of HHS from a mean of 72.33 to 91 at follow-up after a valgus osteotomy procedure for malunion intertrochanteric fracture [3]. In the current study, 30 (37.5%) achieved excellent outcomes, 32 (40.0%) had good outcomes, 14 (17.5%) showed fair outcomes, and four (5.0%) had poor outcomes. These results demonstrate the effectiveness of valgus osteotomy with DHS fixation in restoring hip function and improving quality of life. A study by Kalra et al. treated 20 cases of neglected femoral neck fractures in young adults with valgus intertrochanteric osteotomy, achieving in 90% of patients with an average HHS of 91 [11]. Avascular necrosis occurred in two cases, and 75% of patients reported satisfactory to excellent outcomes after 30 months. Similarly, Varghese et al. also reported excellent to fair outcomes in the majority of cases [5]. These findings align with our results, supporting the efficacy of valgus osteotomy in managing malunion fractures.

Khan et al. observed significant improvements in HHS, with scores increasing from 66.6 to 88 points postoperatively [12]. All patients with united fractures were able to perform one-leg stance, squat, and sit cross-legged, with notable improvements in pain and range of motion. However, two patients experienced implant failure, highlighting the need for careful surgical technique and postoperative monitoring. Bartonícek et al. conducted a prospective study with a mean follow-up of 5.5 years, reporting successful outcomes in 12 patients within four months and delayed union in two cases [13]. One patient required revision surgery due to lost fixation, but the osteotomy ultimately healed. The average HHS improved from 61-83 preoperatively to 92 postoperatively, with no cases of avascular necrosis or osteoarthritis.

These studies collectively suggest that valgus osteotomy with DHS fixation is a reliable and effective treatment for malunion intertrochanteric fractures. It restores limb length, corrects deformity, and improves hip function, offering a cost-effective alternative to THA in selected patients. However, further research with larger sample sizes and longer follow-ups is needed to validate these findings and optimize surgical outcomes.

Limitations

This study has several limitations. It was conducted at a single center with a relatively small sample size, which may limit the generalizability of the findings. The follow-up period of three months may not capture long-term outcomes or complications. Additionally, the study did not compare valgus osteotomy with other surgical techniques, such as THA or intramedullary nailing, which could provide further insights into the optimal management of malunited fractures. Despite these limitations, the results highlight the potential of valgus osteotomy as a viable treatment option for this challenging patient population.

## Conclusions

Valgus osteotomy with DHS fixation demonstrated good and excellent functional outcomes in the majority of the participants. While female and younger patients showed slightly better results, these differences were not statistically significant. Falls were the most common cause of injury, and the procedure achieved high success rates regardless of the mode of injury. These findings support valgus osteotomy with DHS fixation as a reliable treatment for nonunion and malunion trochanteric fractures.
